# A Framework for Characterizing the Multilateral and Directional Interaction Relationships Between PM Pollution at City Scale: A Case Study of 29 Cities in East China, South Korea and Japan

**DOI:** 10.3389/fpubh.2022.875924

**Published:** 2022-05-16

**Authors:** Jianzheng Liu, Hung Chak Ho

**Affiliations:** ^1^School of Public Affairs, Xiamen University, Xiamen, China; ^2^Department of Anaesthesiology, LKS Faculty of Medicine, School of Clinical Medicine, The University of Hong Kong, Hong Kong, Hong Kong SAR, China; ^3^Department of Urban Planning and Design, The University of Hong Kong, Hong Kong, Hong Kong SAR, China

**Keywords:** transboundary air pollution, particulate matter, interaction relationship, China, South Korea, Japan

## Abstract

Transboundary particulate matter (PM) pollution has become an increasingly significant public health issue around the world due to its impacts on human health. However, transboundary PM pollution is difficult to address because it usually travels across multiple urban jurisdictional boundaries with varying transportation directions at different times, therefore posing a challenge for urban managers to figure out who is potentially polluting whose air and how PM pollution in adjacent cities interact with each other. This study proposes a statistical analysis framework for characterizing directional interaction relationships between PM pollution in cities. Compared with chemical transport models (CTMs) and chemical composition analysis method, the proposed framework requires less data and less time, and is easy to implement and able to reveal directional interaction relationships between PM pollution in multiple cities in a quick and computationally inexpensive way. In order to demonstrate the application of the framework, this study applied the framework to analyze the interaction relationships between PM_2.5_ pollution in 29 cities in East China, South Korea and Japan using one year of hourly PM_2.5_ measurement data in 2018. The results show that the framework is able to reveal the significant multilateral and directional interaction relationships between PM_2.5_ pollution in the 29 cities in Northeast Asia. The analysis results of the case study show that the PM_2.5_ pollution in China, South Korea and Japan are linked with each other, and the interaction relationships are mutual. This study further evaluated the framework's validity by comparing the analysis results against the wind vector data, the back trajectory data, as well as the results extracted from existing literature that adopted CTMs to study the interaction relationships between PM pollution in Northeast Asia. The comparisons show that the analysis results produced by the framework are consistent with the wind vector data, the back trajectory data as well as the results using CTMs. The proposed framework provides an alternative for exploring transportation pathways and patterns of transboundary PM pollution between cities when CTMs and chemical composition analysis would be too demanding or impossible to implement.

## Introduction

Transboundary particulate matter (PM) pollution has become an increasingly significant public health issue around the world (1–3). This is because, on one hand, transboundary PM pollution has severe negative impacts on human health due to its associations with respiratory diseases, cardiovascular diseases, birth defects, etc. (4–7), raising considerable public concerns over health and public pressure for government authorities to take actions. On the other hand, transboundary PM pollution is difficult to address, as transboundary PM pollution usually travels across multiple urban jurisdictional boundaries with varying transportation directions at different times driven by synoptic air movement and complex meteorological conditions (1). In other words, PM pollution in a city may interact with PM pollution in adjacent cities, therefore posing a challenge for urban managers in the city to figure out who is potentially polluting whose air, how PM pollution in adjacent cities interact with each other, and with whom they should cooperate in tackling transboundary PM pollution.

Obviously, the interaction relationships between PM pollution in adjacent cities must be examined before taking air pollution mitigation plans and policy measures. An investigation of the interaction relationships between PM pollution in adjacent cities could allow for a better understanding of the transportation and patterns of transboundary PM pollution, which has important implications for air pollution exposome research. Moreover, such an investigation could help inform the formulation of cross-jurisdictional mitigation plans and policy measures. To this end, scholars have developed various approaches suitable for examining the interaction relationships between PM pollution in cities at the city scale (8–10). Generally, these approaches can be divided into two groups. One group is the mechanistic modeling approaches that utilize the mechanism on the physical and chemical processes of air pollutions over time and space to examine the relationships between air pollution in different cities. The other is the statistical modeling approaches that attempt to use statistical methods to explore the relationships without considering the detailed mechanisms.

A typical example of the mechanistic modeling approaches is the Eulerian Chemical Transport Model (CTM). The CTM incorporates a variety of physical schemes and chemical mechanisms to describe the physical and chemical processes of air pollutants over time, including the processes of pollutant emission, transport, chemical transformation, and deposition. Some implementations of the CTMs including Weather Research and Forecasting model coupled with Chemistry (WRF-Chem) (8) and GEOS-Chem (9). These CTMs usually use a Eulerian grid model based on a fixed longitude/latitude coordinate system to describe the space and location. In order to enable the CTMs to simulate the processes of pollutant emission, transport, chemical transformation, and deposition, the CTMs usually require a large amount of spatial data to drive the simulation of various processes. Some of the essential data include emission inventory data to drive the process of pollutant emission, meteorological data to simulate the change of meteorological conditions (e.g., atmospheric pressure, temperature, wind speed and direction, etc.) and drive the process of pollutant transportation (11, 12). The ability of the CTMs in capturing the physical and chemical processes of air pollutants over time and space allows the models to directly link air pollution in the source city to air pollution in the receptor city, thus providing a quantitative and causal explanation of the directional interaction relationships between air pollution in multiple cities.

Although CTMs are able to provide high-quality characterization of interaction relationships between air pollution in multiple cities, the CTMs are cumbersome, demanding, and sometimes impossible to implement. First, the CTMs require a large amount of high-quality spatial data, and these data are usually not available in underdeveloped areas. Even in developed regions, the quality of data heavily affects the accuracy of analysis results and may cause huge uncertainties (6, 11, 13–16). Second, the execution of the models is costly both in terms of finance and time. CTMs are usually run in expensive high-performance computing clusters, and it usually takes months to complete a typical CTM run. Moreover, the technical complexity and difficulty of running CTMs are high (1). Generally, only experts who are trained in numerical simulation in the field of atmospheric science are capable to configure, debug and run a CTM.

The statistical modeling approaches do not rely on the detailed mechanism of physical and chemical processes of air pollutants. This group of approaches usually infer the interaction relationships based on assumptions. In other words, this group of approaches can only indicate association (or correlation), but not causation. Chemical composition analysis, for example, is developed based on the assumption that the chemical composition of air pollutants is unique in different places, and the unique chemical composition of air pollutants represents the unique identity of that place. The more similar the chemical composition of air pollutants in one city to the chemical composition of air pollutants in the other city, the more likely the air pollution in the two cities were associated with each other (17). For example, the ratio of two isotopes (^206^Pb/^207^Pb) was used to infer the interaction relationship between Pb deposition in Singapore and other countries in Southeast Asia (18). The method of chemical composition analysis is not able to determine the direction of the interaction relationship. Fortunately, with the help of radiometric dating used in paleoenvironmental studies (19), the time of samples can be estimated, which ascertains the chronology and further determines the direction of the interaction relationship.

Chemical composition analysis is a relatively convincing statistical tool for inferring interaction relationships between air pollution in cities. However, the assumption on the uniqueness of the chemical composition of air pollutants across different cities is doubtful because the chemical composition of air pollutants in a place changes over time (20). Moreover, this method requires laborious sample collection processes in various sampling points. The accuracy of chemical composition analysis depends on the number of samples. Furthermore, the spatial extent of chemical composition analysis is strongly limited by the locations of the sampling sites (1).

In summary, existing methods are able to examine the interaction relationships between PM pollution in multiple cities, but are strongly limited by the availability of data and costs in terms of time and finance. In cities and regions where there is no data available, or only have limited financial resources, it is impossible and too expensive to implement the methods mentioned above. In fact, most of developing countries and under-developed areas such as South Asia and Central Asia do not have proper emission inventory data customized for running CTMs in local areas (most of existing emission inventory data are usually developed at a very coarse spatial resolution for global or continental-scale simulations) (17, 21–24). Nor did these areas conducted large-scale sampling campaigns for chemical composition analyses (17). This is because the development of such a customized emission inventory data and large-scale sampling campaigns requires persistent financial input and collaboration of hundreds of scientists. Therefore, a simple and easy-to-implement method, that requires less data, less time and less labor to examine the interaction relationships between PM pollution in multiple cities, would therefore be useful.

This research aims to provide an alternative statistical analysis framework for characterizing directional interaction relationships between PM pollution in multiple cities when CTMs and chemical composition analysis would be too demanding or impossible to implement. This analysis framework integrates the cross-correlation function with Granger causality test. It requires only PM measurement data, which can be obtained from existing air quality monitoring network or low-cost air quality sensors. It is easy to implement and is able to reveal directional interaction relationships between PM pollution in multiple cities in a quick and computationally inexpensive way.

In the following section, this study introduces the analysis framework in details. Then a case study of 29 cities in East China, South Korea and Japan is carried out to illustrate the analysis framework. The 29 cities in Northeast Asia are selected as the study area in that cities in Northeast Asia have been suffering from severe transboundary PM pollution for decades, raising considerable public concern; moreover, CTMs have been used to simulate the transboundary PM pollution in Northeast Asia and these CTM simulation results could be used to verify the analysis results produced by the framework proposed in this study. In the concluding section, this paper summarizes the advantages of the framework and its limitations.

## The Framework For Characterizing The Multilateral and Directional Interaction Relationships Between Pm Pollution At City Scale

The proposed framework consists of two steps. The first step is to compute the strengths of potential interaction relationships between PM pollution in adjacent cities using the cross-correlation function. The second step is to determine the directions of the interaction relationships using Granger causality tests. Each step is introduced in detail below.

### The Cross-Correlation Function

The strength of a potential interaction relationship between PM pollution in two adjacent cities is measured using a statistical measure called time lag-adjusted Pearson correlation coefficient. The time lag-adjusted Pearson correlation coefficient is calculated using cross correlation method (25–27).

First, the Pearson correlation coefficients between two PM concentration time series in each pair of cities are calculated at continuously varying time lags. This can be mathematically described in the following equation:


(1) $$\begin{array}{l} P\left(\tau \right)=Corr\left(X_{1}\left(t\right),\;X_{2}\left(t-\tau \right)\right), \end{array}$$

where *P*(τ) is the Pearson correlation coefficient between two PM concentration time series at a specific time lag value τ, and *X*_1_ and *X*_2_ are the two PM concentration time series in the pair of cities.

The value range of the time lag τ is set according to the patterns of synoptic cycles of the study area. For example, in Northeast Asia, the synoptic meteorological system usually influences the air quality in the region on a weekly basis (28), therefore the time lag value τ in the Equation (1) is set to vary between the past 7 days (−168 h) and subsequent 7 days (+168 h).

Then, the maximum Pearson correlation coefficient among all the coefficients calculated in the first step is identified, as described in Equation (2).


(2) $$\begin{array}{l} P_{\max}=\max\left(P\left(\tau \right)\right), \end{array}$$

*P*_max_ is the maximum correlation coefficient which indicates the strength of the interaction relationship between PM pollution in the pair of cities.

Two tests of significance are performed to ensure the results are statistically significant. The first test is the significance test of the correlation coefficient which is used to test whether the calculated Pearson's correlation coefficient is significantly different from zero. The second test is the significance test of the difference between two correlation coefficients using Fisher's r-to-z transformation (29, 30), which is used to examine whether the maximum correlation coefficient (*P*_max_) is significantly larger than the correlation coefficient without the time lag (*P*(0)). If *P*_max_ is significantly larger than *P*(0), the observed difference between the two coefficients is not due to random chance.

### The Granger Causality Test

After the strength of the interaction relationship between PM pollution in the pair of cities is calculated using the cross-correlation function and the significance of the interaction relationship is confirmed by statistical tests in the first step, the next step is to determine the direction of the interaction relationship between PM pollution in the pair of cities.

First, the time lag that generates the maximum correlation coefficient *P*_max_ is identified, as shown in Equation (3).


(3) $$\begin{array}{l} T_{delay}=argmax_{\tau }\left(P\left(\tau \right)\right), \end{array}$$

The sign of *T*_*delay*_ shows the potential temporal order of the two PM concentration time series in the pair of cities, which suggests the potential direction of the interaction relationship.

Then, Granger causality tests are applied to confirm the potential direction. The Granger causality test is a statistical hypothesis test for inferring causal influences between variables based on temporal precedence (31, 32). The rationale of the Granger causality test is that, given an autoregressive model that predict the future values of the PM concentration in a city (*X*_2_) based on the past values of *X*_2_, if adding the lagged values of the PM concentration in the other city (*X*_1_) into the model can better model *X*_2_, then *X*_1_ is said to Granger-cause *X*_2_. In this way, we can confirm the direction of influence between *X*_1_ and *X*_2_. The advantage of the Granger causality test over correlation analysis is that it can remove spurious correlations of the PM time series and therefore reduce the risk of reporting false associations with wrong directions (32). Although Granger causality cannot directly reflect the real physical causal chains, it provides relatively convincing statistical evidence for inferring causality without requiring additional data.

**Figure 1:**
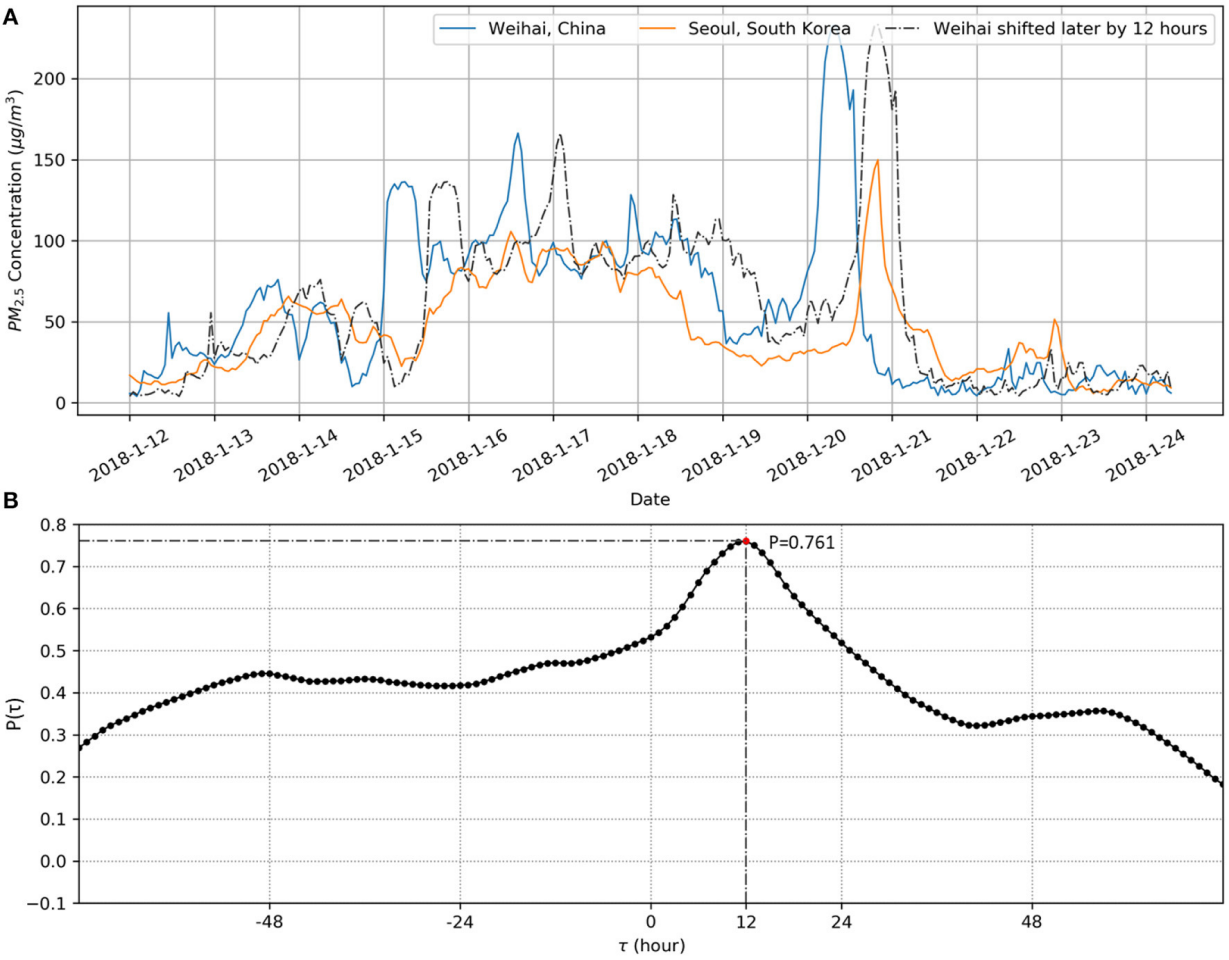
The calculation of the time lag-adjusted Pearson correlation coefficient. **(A)** An illustration of the time-lagged correlation between PM_2.5_ concentration time series in Weihai, China and Seoul, South Korea. The dashed line shows the aligned PM_2.5_ time series in Weihai, which is shifted later by 12 h to attain the best alignment with the PM_2.5_ time series in Seoul. **(B)** The Pearson correlation coefficients [*P*(τ)] between the two PM_2.5_ concentration time series during January 12–24, 2018 in Weihai and Seoul at varying time lags (τ).

Figure 1 gives an illustration of the calculation process of the time lag-adjusted Pearson correlation coefficient using fine particulate matter (PM_2.5_) measurement data in two cities of Northeast Asia. As shown in Figure 1A, the two PM_2.5_ concentration time series in Weihai, China and Seoul, South Korea are best aligned when Weihai's PM_2.5_ time series is shifted later by 12 h. The Pearson correlation coefficient calculated at the time lag of 12 is the maximum Pearson correlation coefficient among all the coefficients calculated at varying time lags (Figure 1B). Statistical tests confirm the statistical significance of the correlation coefficient. Then, the time lag that generates the maximum correlation coefficient can be identified as −12. Lastly, Granger causality test confirms that the direction of influence in the interaction relationship between PM_2.5_ pollution in Weihai and Seoul is from Weihai to Seoul, suggesting that the PM_2.5_ pollution in Weihai may have an impact on the PM_2.5_ pollution in Seoul during January 2018.

## A Case Study of 29 Cities In East China, South Korea and Japan

### Data

**Figure 2:**
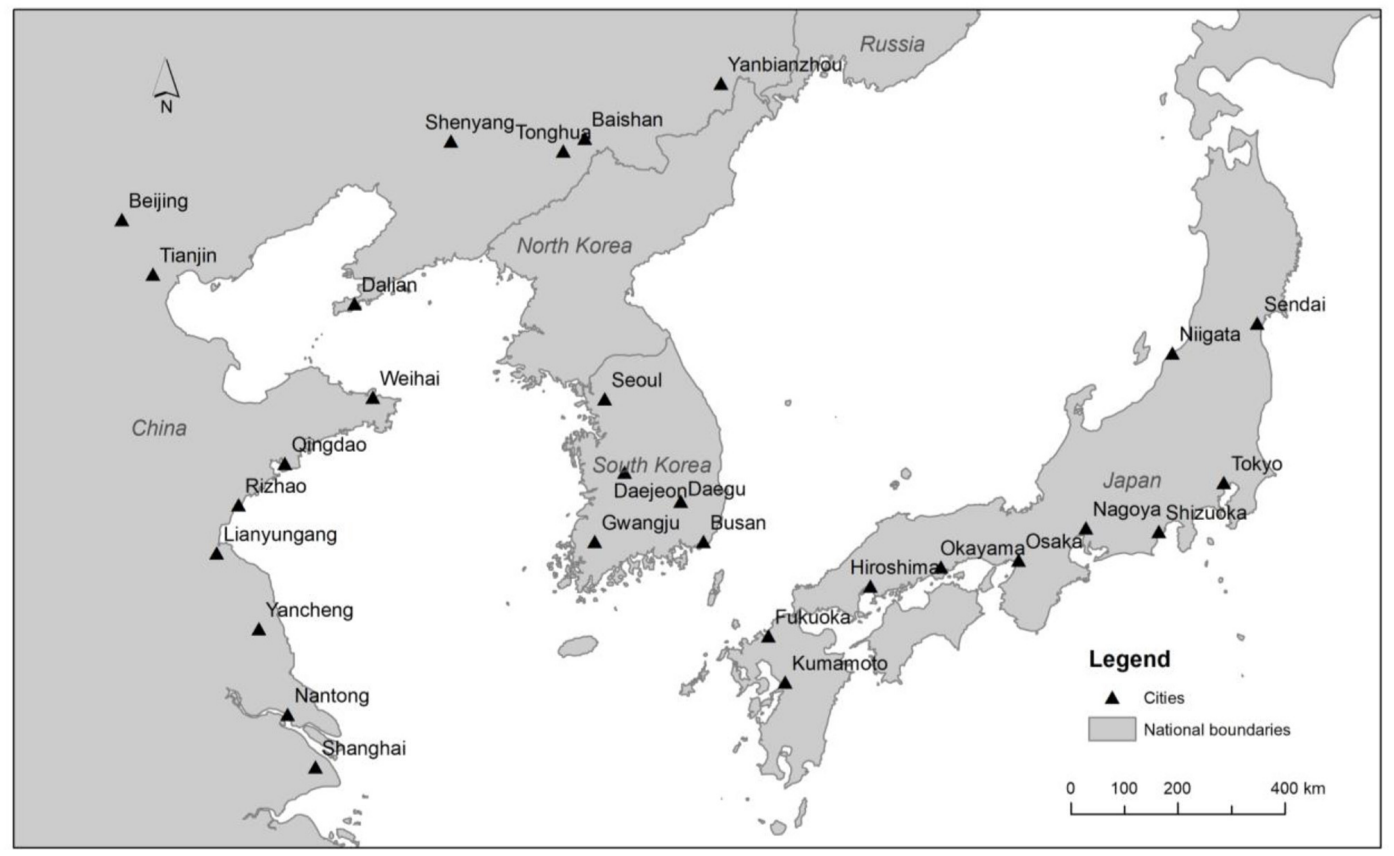
Study area.

This study collected a full year of hourly PM_2.5_ measurement data in 2018 from 29 major cities with a population of over 1 million in Northeast Asia from the environmental monitoring agencies in China, South Korea and Japan. The 29 cities include 14 cities in East China (Beijing, Tianjin, Dalian, Shenyang, Tonghua, Baishan, Yanbianzhou, Weihai, Qingdao, Rizhao, Lianyungang, Yancheng, Nantong and Shanghai), 5 cities in South Korea (Seoul, Daejeon, Daegu, Gwangju and Busan) and 10 cities in Japan (Tokyo, Niigata, Sendai, Shizuoka, Nagoya, Osaka, Okayama, Hiroshima, Fukuoka and Kumamoto). Figure 2 shows the study area.

This study conducted a comprehensive data quality check to remove problematic data points, including implausible zeros, duplicated data records and missing measurements. Extremely high hourly PM_2.5_ measurements (>1,000 μg/m^3^) are considered as outliers and therefore removed. After the data quality check, the hourly PM_2.5_ measurements at all monitoring stations in each city were averaged to generate an hourly PM_2.5_ time series for that city. As China Standard Time (UTC+08:00) is 1 h earlier than Japan Standard Time and Korean Standard Time (UTC+09:00), the timestamps for all PM_2.5_ time series in South Korea and Japan were adjusted to China Standard Time for the convenience of data analysis.

In addition, this study used a weather reanalysis dataset developed by NASA to draw wind vector maps at 50 m above the surface in 2018 in Northeast Asia. The dataset is produced based on atmospheric, land, and ocean observations from satellites, aircraft, and ships in the NASA's project of Modern-Era Retrospective analysis for Research and Applications version 2 (MERRA-2) (33).

### Results

In order to show the temporal variation of the interaction relationships between PM_2.5_ pollution in the 29 cities in East China, South Korea and Japan, this study performs the analysis using the framework on a monthly basis.

**Figure 3:**
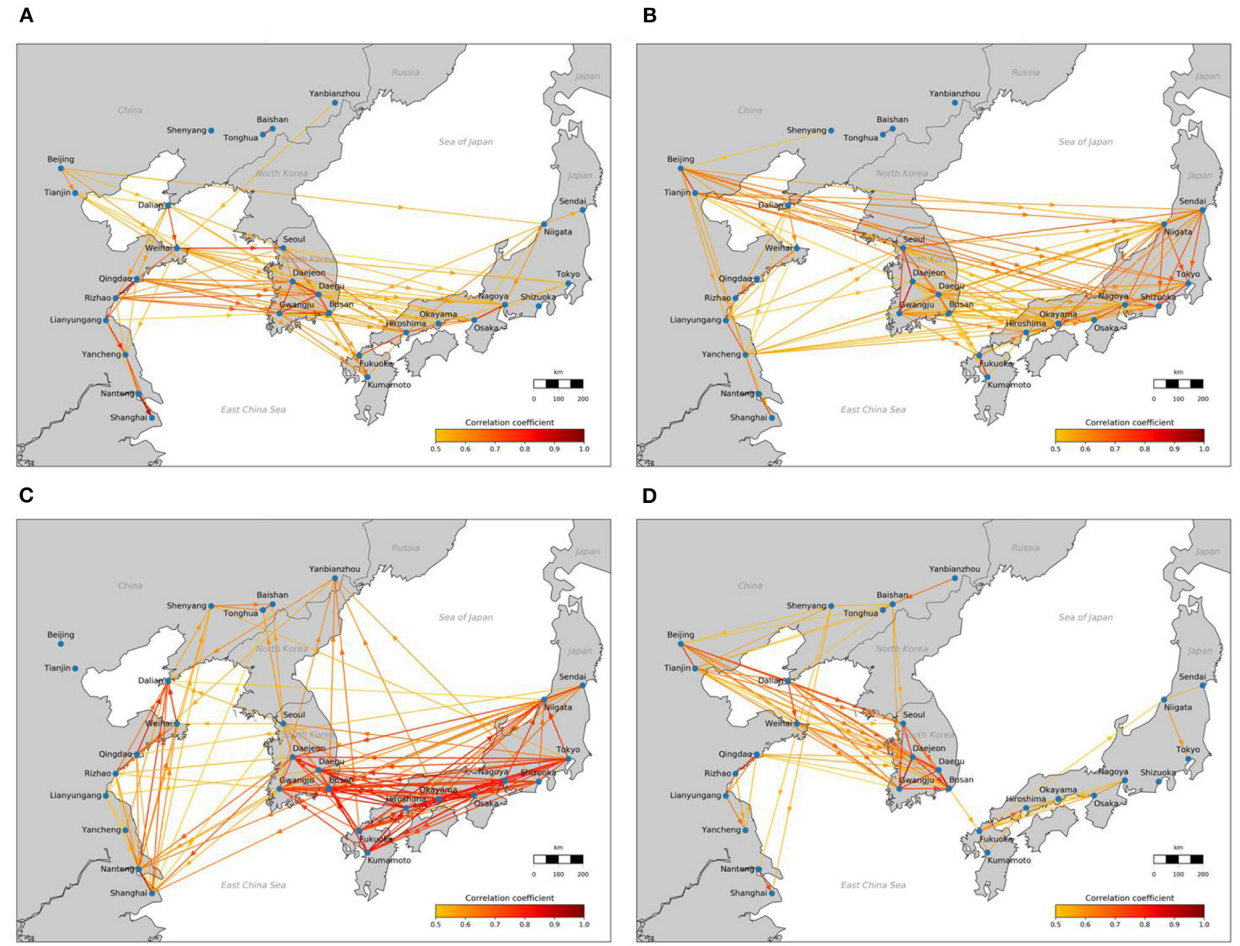
The multilateral and directional interactions between PM_2.5_ pollution in the 29 cities in East China, South Korea and Japan, in **(A)** January, **(B)** April, **(C)** July, and **(D)** October 2018.

The analysis results in January, April, July and October 2018 were calculated and visualized in maps (see Figure 3). As shown in Figure 3, each line connecting two cities indicates that there is a significant interaction relationship between the PM_2.5_ pollution in the two cities. The colors of the lines indicate the strengths of the interaction relationship. The arrows in the lines show the temporal order of the corresponding PM_2.5_ time series, which suggest the prevailing transportation directions of air parcels.

The visualizations in Figure 3 show that the interaction relationships between the PM_2.5_ time series of pairs of cities in East China, South Korea and Japan are significant and strong in all four seasons. In January, April and October, the PM_2.5_ time series in Japan lagged behind the PM_2.5_ time series in South Korea, which in turn lagged behind the PM_2.5_ time series in East China; this suggests that the PM_2.5_ pollution probably flows from China to South Korea to Japan. Conversely, in July, the PM_2.5_ pollution probably flows from Japan to South Korea to China. In summary, the results demonstrate the strong and significant multilateral and directional interactions between PM_2.5_ pollution in cities in Northeast Asia.

The results on the multilateral and directional interaction relationships between PM_2.5_ pollution in the 29 cities show how PM_2.5_ pollution in China, South Korea and Japan interact with each other, which suggests that transboundary PM_2.5_ pollution in China, South Korea and Japan are linked with each other. It is therefore recommended that various stakeholders such as the general public, media and government agencies in China, South Korea and Japan be made aware of that a cooperative relationship and mutual support among all stakeholders are important for building a broader coalition in mitigating transboundary air pollution.

## Evaluation

This study used three data sets to verify the results produced by the framework on the interaction relationships between PM_2.5_ pollution in the 29 cities in Northeast Asia. The first two data sets are the wind vector data and calculated trajectories of air mass movement. The third data set is the data extracted from existing literature that adopted CTMs to quantify the relationships.

### Evaluation Using the Wind Vector Data and Back Trajectories

**Figure 4:**
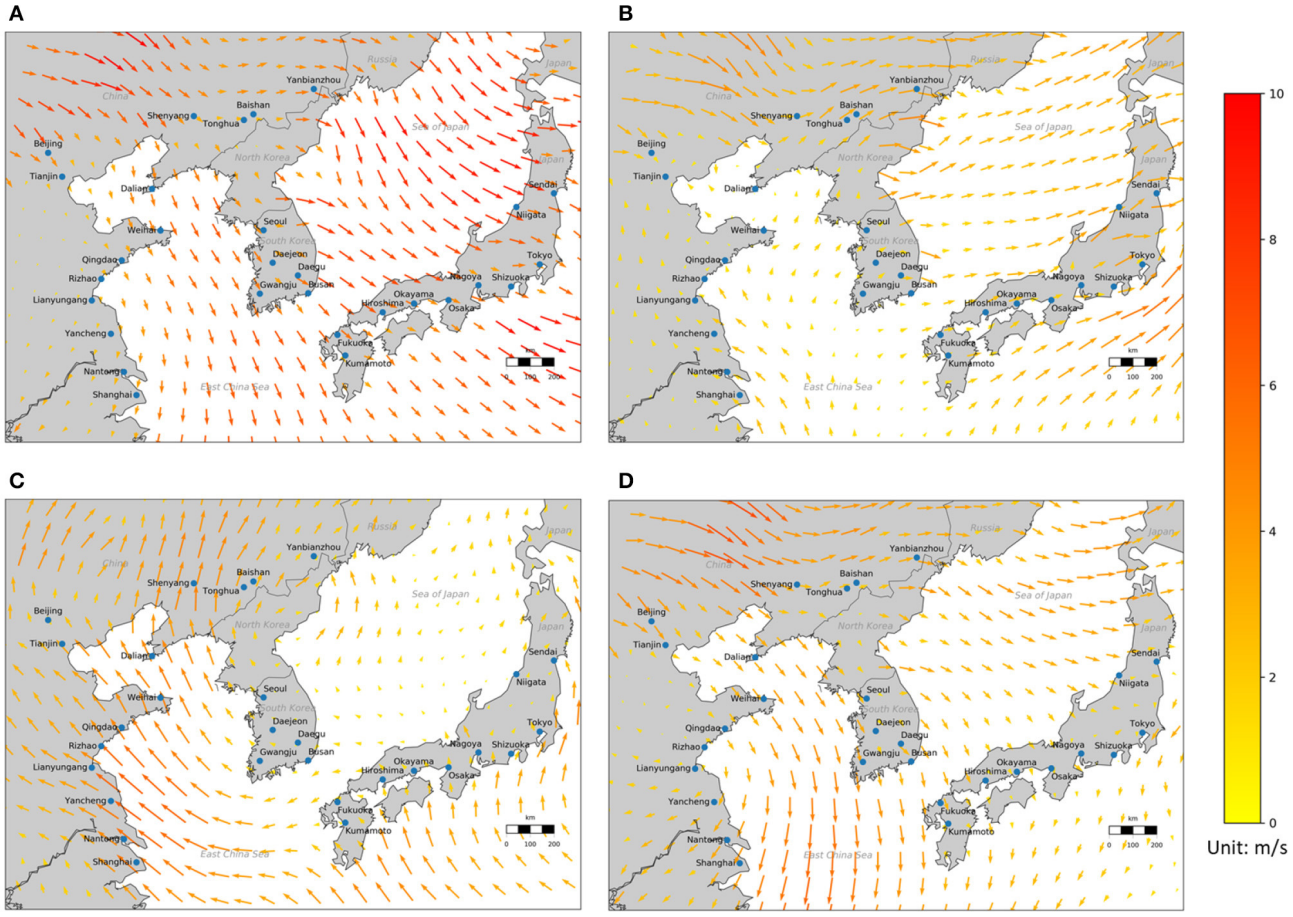
Monthly-averaged wind vectors at 50 m above the surface in **(A)** January, **(B)** April, **(C)** July, and **(D)** October 2018 in Northeast Asia. Unit: m/s.

Figure 4 shows four monthly-averaged wind vector maps in January, April, July and October 2018 at 50 m above the surface in Northeast Asia, which were drawn using a MERRA-2 weather reanalysis data developed by NASA (33). As shown in Figures 3 and 4, the directions of the interaction relationships between PM_2.5_ pollution in the 29 cities match the wind vectors very well.

In addition, this study calculated backward trajectories of the air masses reaching Seoul, South Korea based on the Global NOAA-NCEP/NCAR reanalysis meteorological data for the year of 2018 using NOAA's Hybrid Single Particle Lagrangian Integrated Trajectory (HYSPLIT, version 4) model. The HYSPLIT model is able to trace air parcels' paths back in time and space and indicate where the air parcels have been before they reach the receptor site (34). Each trajectory had a run time of 96 h with 3 h time intervals. The Python package Matplotlib and the R package *openair* (http://www.openair-project.org/) developed by Carslaw and Ropkins (35) were used to visualize the back trajectories produced by the HYSPLIT trajectory model.

**Figure 5:**
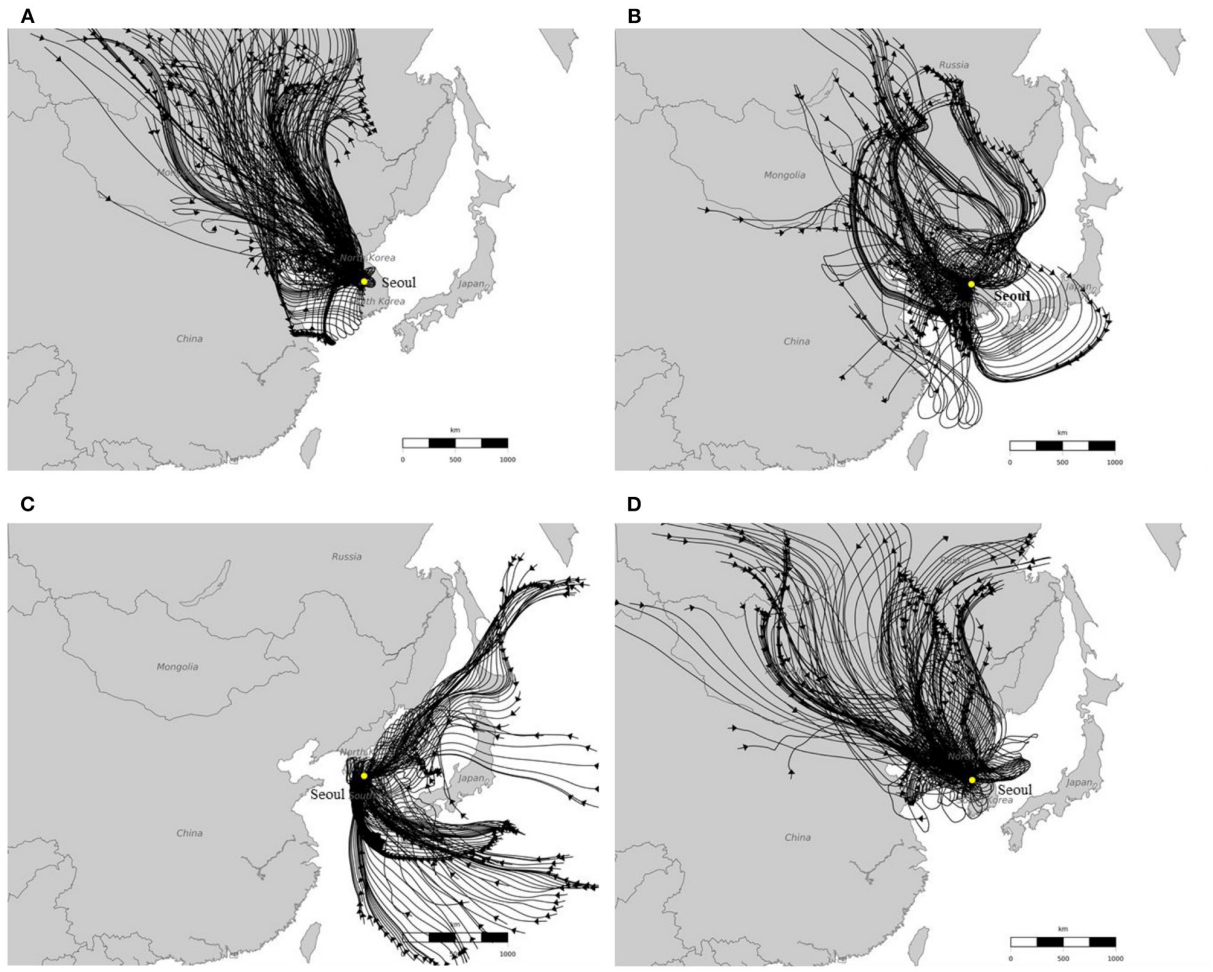
The 96-h HYSPLIT back trajectories with Seoul, South Korea as the receptor site in **(A)** January, **(B)** April, **(C)** July, and **(D)** October 2018.

Figure 5 shows the results of the 96-h back trajectories centered on Seoul, South Korea in January, April, July and October 2018. As Figure 5 shows, in January, April and October 2018, the air parcels traveled from China to South Korea, while in July 2018, the air parcels traveled from Japan to South Korea. It can be further inferred from Figure 5 that, the air parcels continued to travel from South Korea to Japan in January, April and October 2018, while in July 2018 the air parcels continued to travel from South Korea to China. It can be seen that the results of back trajectory simulations and wind vectors are consistent with the results on the directional interactions between PM_2.5_ pollution in the 29 cities of Northeast Asia produced using the framework proposed in this study.

Figures 4 and 5 also suggest that the seasonal pattern of the interaction relationships between PM_2.5_ pollution in the 29 cities of East China, South Korea and Japan is probably driven by the atmospheric circulation, particularly the westerlies and the East Asian monsoon, which usually brings south-eastern winds in summer and north-eastern winds in winter (36).

### Evaluation Using the Data Produced by CTMs in the Literature

As introduced in the section of introduction, CTMs are able to produce high-quality analysis results on the interaction relationships between air pollution in multiple cities. Fortunately, a handful of modeling studies using CTMs have been carried out to study the interaction relationships between PM pollution in China, South Korea and Japan (37–41). Although the interaction relationships are analyzed at national/regional scale and the results are calculated at national/regional level in these studies, the results of these studies are helpful and can be used to verify the analysis results in this study.

Table 1 shows a list of studies that quantified the interaction relationships between PM pollution in China, South Korea and Japan using CTMs. The table listed the pollutants modeled, the time period simulated, the data figures that quantified the interaction relationships between PM pollution in China, South Korea and Japan. For example, in the study by Kajino et al. (40), the CTM modeling results showed that in 2006, China contributed 76.5 Gg and 364 Gg of total sulfate deposition to South Korea and Japan, respectively; South Korea contributed 44.1 Gg and 60.8 Gg of total sulfate deposition to China and Japan, respectively; and Japan contributed 31.8 Gg and 16.4 Gg of total sulfate deposition to China and South Korea, respectively. These results produced by CTMs clearly show that the interaction relationships between PM pollution in China, South Korea and Japan are significant and mutual.

**Table 1:** A list of CTM studies and their quantified interaction relationships between PM pollution in China, Korea and Japan.

**Entry**	**Time period**	**Pollutant**	**Unit**	**Contribution of China to Korea**	**Contribution of China to Japan**	**Contribution of Korea to China**	**Contribution of Korea to Japan**	**Contribution of Japan to China**	**Contribution of Japan to Korea**
LTP (37)	2017	PM_2.5_	Percentage	32%	25%	1.9%	8.2%	0.80%	1.5%
Li et al. (38)	2010	PM_10_	μg/m^3^	11.2	2.80	0.100	0.600	0.000	0.200
Kajino et al. (39)	2006	Total nitrate deposition	Gg	19.3	74.8	7.69	13.3	0.0590	1.62
Kajino et al. (40)	2002	Total sulfate deposition	Gg	76.5	364	44.1	60.8	31.8	16.4
Lin et al. (41)	2001	total nitrogen deposition	Percentage	39%	21%	2.6%	15%	0.50%	4.6%

*Note that nitrate and sulfate are components of particulate matter (PM). The percentages are calculated as the ratio between the amount of pollutants one country contributed to the other country and the total amount of pollutants the other country received. “Korea” refers to South Korea except that “Korea” refers to North Korea and South Korea in the study by Lin et al. (41)*.

**Figure 6:**
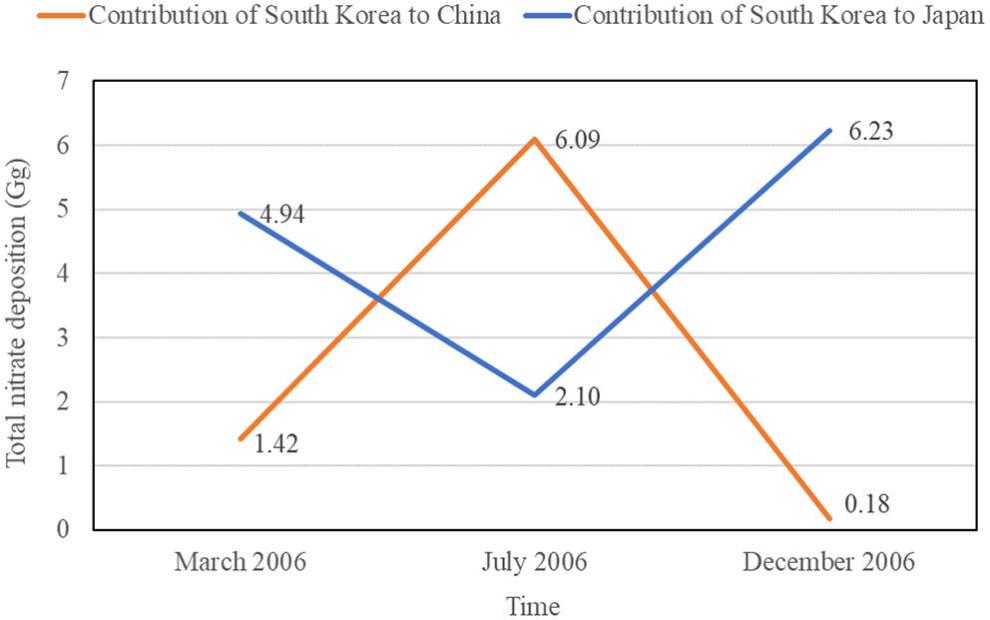
The contribution of South Korea to China and Japan in terms of total nitrate deposition in March, July and December 2006 in the study by Kajino et al. (39).

In addition, the study by Kajino et al. (39) quantified the interaction relationships of total nitrate deposition in March, July, and December 2006, respectively, which enable a seasonal comparison. As shown in Figure 6, from March to July, the contribution of South Korea to the total nitrate deposition in China increased, but the contribution of South Korea to Japan decreased; from July to December, the contribution of South Korea to the total nitrate deposition in China decreased, but the contribution of South Korea to Japan increased. Obviously, the directions of influence in the results produced by CTM are consistent with the directions identified in the analysis results produced by the framework in this study.

## Advantages and Limitations of The Framework

The proposed framework in this study has several advantages in examining the interaction relationships between PM pollution in cities compared with CTMs and chemical composition analysis. As shown in Table 2, the framework requires much less data than CTMs. The framework only needs the PM measurement data of the cities of interest which can be obtained from existing air quality monitoring network or low-cost air quality sensors. The CTMs, however, not only require the PM measurement data of the cities to evaluate and calibrate the model, but also require emission inventory and meteorological data to drive the simulation of the processes of pollutant emission, transport, chemical transformation, and deposition.

**Table 2:** Comparison between the framework proposed in this study, CTMs and chemical composition analysis.

**Method**	**Type of approach**	**Quality of results**	**Data required**	**Time required**	**Technical difficulty**
CTMs (e.g., GEOS-Chem)	Mechanistic modeling approach	Able to establish causal relationships between PM pollution and quantify the relationship	1. Meteorological data; 2. Emission inventory data; 3. Pollutant measurement data	Several months for a domain such as Northeast Asia	High, difficult to learn and execute
Chemical composition analysis	Statistical modeling approach	Able to reveal relationships between PM pollution in cities	1. Chemical composition data of pollutant	Usually takes several months even years	Medium
The framework proposed in this study	Statistical modeling approach	Able to reveal relationships between PM pollution in cities and determine its direction of influence	1. Pollutant measurement data	Several hours	Low, easy to learn and implement

The framework also costs much less time to implement, and is easy to execute. It usually takes a few hours to apply this framework using the PM measurement data. But, for a CTM, it usually takes months to execute. For example, a CTM such as the GEOS-Chem model is used to simulate the PM pollution in East Asia. The simulations are carried out in a nested domain at a horizontal resolution of 1/2° latitude by 2/3° longitude over the East Asia. The nested domain is embedded in a global chemical transport simulation at a horizontal resolution of 4° latitude by 5° longitude, which provides initial and boundary conditions for the nested domain. The computer to run the model has 128 gigabyte computer memory and 2 Intel Xeon processors with each processor having 16 cores (Intel Xeon Gold 5218 CPU). Then it needs approximately two months to complete the simulations. As for chemical composition analysis, it usually takes several months even years to collect a sufficient number of samples. Moreover, when the samples are collected, the samples have to be analyzed using expensive devices to obtain the detailed chemical compositions.

In summary, compared with CTMs and chemical composition analysis, the proposed framework in this study provides a simple but valid and easy-to-implement method, that requires less data and less time, to examine the interaction relationships between PM pollution in multiple cities. The framework provides an alternative for exploring the transportation pathways and potential source areas when CTMs and chemical composition analysis would be too demanding or impossible to implement.

The proposed framework in this study has two limitations. The first limitation is that it cannot establish causal interaction relationships between PM pollution in multiple cities the way the CTMs are able to do (Table 2). As the case study shows, the analysis results produced by the framework show there exist significant multilateral and directional interaction relationships between PM_2.5_ pollution in the 29 cities in Northeast Asia. These interaction relationships show that PM_2.5_ pollution in China, South Korea and Japan interacted with each other. However, these associated relationships with directions can only suggest that there may exist probable causal relationships that PM_2.5_ pollution in a city causing the PM_2.5_ pollution in another city, but cannot be certain that these interaction relationships between PM_2.5_ pollution have causal linkages. The second limitation is that the framework is not able to quantify the interaction relationships between PM pollution in cities as the CTMs are able to do (Table 2). In other words, the framework can answer whether there is a significant interaction relationship between PM pollution in two cities and what is the direction of influence in the relationship, but cannot answer to what extent the PM pollution in one city affects the PM pollution in the other city and how much PM pollution is transported to the other city.

## Data Availability Statement

Publicly available datasets were analyzed in this study. The dataset of hourly PM_2.5_ measurement data can be found in the websites of the environmental monitoring agencies in China (https://air.cnemc.cn:18007/), South Korea (http://www.airkorea.or.kr/) and Japan (http://soramame.taiki.go.jp/). The weather reanalysis dataset used to draw wind vector maps in this study is available from NASA Modern-Era Retrospective analysis for Research and Applications version 2 dataset (MERRA-2) website (https://gmao.gsfc.nasa.gov/reanalysis/MERRA-2/data_access/).

## Author Contributions

JL conceived the study, analyzed the data, and wrote the paper. HH analyzed the data. Both authors contributed to the article and approved the submitted version.

## Funding

This research was supported by the Social Science Research Base Program of Fujian at the Research Center of Public Service Quality of Xiamen University (Grant No. FJ2020JDZ006) and the National Natural Science Foundation of China (Grant No. 42101199).

## Conflict of Interest

The authors declare that the research was conducted in the absence of any commercial or financial relationships that could be construed as a potential conflict of interest.

## Publisher's Note

All claims expressed in this article are solely those of the authors and do not necessarily represent those of their affiliated organizations, or those of the publisher, the editors and the reviewers. Any product that may be evaluated in this article, or claim that may be made by its manufacturer, is not guaranteed or endorsed by the publisher.

## Abbreviations

PM: particulate matter
PM_2.5_: fine particulate matter
CTM: Chemical Transport Model
WRF-Chem: Weather Research and Forecasting model coupled with Chemistry
NASA: the United States National Aeronautics and Space Administration
MERRA-2: Modern-Era Retrospective analysis for Research and Applications version 2
NOAA: the United States National Oceanic and Atmospheric Administration
HYSPLIT: Hybrid Single-Particle Lagrangian Integrated Trajectory Model
LTP: Joint Research Project for Long–range Transboundary Air Pollutants in Northeast Asia.

## References

[1] Liu J Li J Yao F. Source-receptor relationship of transboundary particulate matter pollution between China, South Korea and Japan: approaches, current understanding and limitations. Crit Rev Environ Sci Technol. (2021) 2021:1–25. 10.1080/10643389.2021.1964308

[2] Jiao Y Su M Ji C Yang S Zhang P. How to design fully cooperative policies to abate transboundary air pollution between two highly asymmetric regions: An abnormal incrementalism analysis. J Clean Prod. (2021) 278:124042. 10.1016/j.jclepro.2020.124042

[3] Halkos G Tsilika K. Understanding transboundary air pollution network: Emissions, depositions and spatio-temporal distribution of pollution in European region. Resour Conserv Recycl. (2019) 145:113–23. 10.1016/j.resconrec.2019.02.014

[4] Shen WT Yu X Zhong SB Ge HR. Population health effects of air pollution: fresh evidence from china health and retirement longitudinal survey. Front Public Health. (2021) 9:12. 10.3389/fpubh.2021.779552 35004584 PMC8733201

[5] Feng S Gao D Liao F Zhou F Wang X. The health effects of ambient PM_2.5_ and potential mechanisms. Ecotoxicol Environ Saf. (2016) 128:67–74. 10.1016/j.ecoenv.2016.01.030 26896893

[6] Guo Y Feng N Christopher SA Kang P Zhan FB Hong S. Satellite remote sensing of fine particulate matter (PM_2.5_) air quality over Beijing using MODIS. Int J Remote Sens. (2014) 35:6522–44. 10.1080/01431161.2014.95824530857313

[7] Zhao Y Kong D Fu J Zhang Y Chen Y Liu Y . Increased risk of hospital admission for asthma in children from short-term exposure to air pollution: case-crossover evidence from Northern China. Front Public Health. (2021) 9, 798746. 10.3389/fpubh.2021.798746 PMC8718688 34976938

[8] Grell GA Peckham SE Schmitz R McKeen SA Frost G Skamarock WC . Fully coupled “online” chemistry within the WRF model. Atmos Environ. (2005) 39:6957–75. 10.1016/j.atmosenv.2005.04.027

[9] Henze DK Hakami A Seinfeld JH. Development of the adjoint of GEOS-Chem. Atmos Chem Phys. (2007) 7:2413–33. 10.5194/acp-7-2413-2007

[10] Inomata Y Ohizumi T Take N Sato K Nishikawa M. Transboundary transport of anthropogenic sulfur in PM_2.5_ at a coastal site in the Sea of Japan as studied by sulfur isotopic ratio measurement. Sci Total Environ. (2016) 553:617–25. 10.1016/j.scitotenv.2016.02.139 26970199

[11] Zhong M Saikawa E Liu Y Naik V Horowitz LW Takigawa M . Air quality modeling with WRF-Chem v35 in East Asia: sensitivity to emissions and evaluation of simulated air quality. Geosci Model Dev. (2016) 9:1201–18. 10.5194/gmd-9-1201-2016

[12] Kakosimos K Assael M Lioumbas J Spiridis A. Atmospheric dispersion modelling of the fugitive particulate matter from overburden dumps with numerical and integral models. Atmos Pollut Res. (2011) 2:24–33. 10.5094/APR.2011.004

[13] Ding J Miyazaki K Johannes VR Mijling B Kurokawa JI Cho S . Intercomparison of NOx emission inventories over East Asia. Atmos Chem Phys. (2017) 17:10125–41. 10.5194/acp-17-10125-2017

[14] Li M Liu H Geng G Hong C Liu F Song Y . Anthropogenic emission inventories in China: a review. Natl Sci Rev. (2017) 4:834–66. 10.1093/nsr/nwx150

[15] Zhu S Kinnon MM Shaffer BP Samuelsen GS Brouwer J Dabdub D. An uncertainty for clean air: air quality modeling implications of underestimating VOC emissions in urban inventories. Atmos Environ. (2019) 211:256–67. 10.1016/j.atmosenv.2019.05.019

[16] Crippa M Janssens-Maenhout G Guizzardi D Van Dingenen R Dentener F. Contribution and uncertainty of sectorial and regional emissions to regional and global PM_2.5_ health impacts. Atmos Chem Phys. (2019) 19:5165–86. 10.5194/acp-19-5165-2019

[17] Chen QQ Taylor D. Transboundary atmospheric pollution in Southeast Asia: current methods, limitations and future developments. Crit Rev Environ Sci Technol. (2018) 48:997–1029. 10.1080/10643389.2018.1493337

[18] Chen M Boyle EA Switzer AD Gouramanis C. A century long sedimentary record of anthropogenic lead (Pb), Pb isotopes and other trace metals in Singapore. Environ Pollut. (2016) 213:446–59. 10.1016/j.envpol.2016.02.040 26967352

[19] Kuwae M Tsugeki NK Agusa T Toyoda K Tani Y Ueda S . Sedimentary records of metal deposition in Japanese alpine lakes for the last 250 years: recent enrichment of airborne Sb and In in East Asia. Sci Total Environ. (2013) 442:189–97. 10.1016/j.scitotenv.2012.10.037 23178779

[20] Thiemens MH. History and applications of mass-independent isotope effects. Annu Rev Earth Planet Sci. (2006) 34:217–62. 10.1146/annurev.earth.34.031405.125026

[21] Anjum MS Ali SM Imad-ud-din M Subhani MA Anwar MN Nizami A-S . An emerged challenge of air pollution and ever-increasing particulate matter in Pakistan; a critical review. J Hazard Mater. (2021) 402:123943. 10.1016/j.jhazmat.2020.123943 33254830

[22] Li M Zhang Q Kurokawa JI Woo JH He K Lu Z . MIX: a mosaic Asian anthropogenic emission inventory under the international collaboration framework of the MICS-Asia and HTAP. Atmos Chem Phys. (2017) 17:935–63. 10.5194/acp-17-935-2017

[23] Ohara T Akimoto H Kurokawa J Horii N Yamaji K Yan X . An Asian emission inventory of anthropogenic emission sources for the period 1980-2020. Atmos Chem Phys Discuss. (2007) 7:6843–902. 10.5194/acpd-7-6843-2007

[24] Janssens-Maenhout G Crippa M Guizzardi D Dentener F Muntean M Pouliot G . HTAP_v2.2: a mosaic of regional and global emission grid maps for 2008 and 2010 to study hemispheric transport of air pollution. Atmos Chem Phys. (2015) 15:11411–32. 10.5194/acp-15-11411-2015

[25] Liu J Li W Wu J. A framework for delineating the regional boundaries of PM_2.5_ pollution: A case study of China. Environ Pollut. (2018) 235:642–51. 10.1016/j.envpol.2017.12.064 29331897

[26] Liu J Li W Wu J Liu Y. Visualizing the intercity correlation of PM_.5_ time series in the Beijing-Tianjin-Hebei region using ground-based air quality monitoring data. PLoS ONE. (2018) 13:e0192614. 10.1371/journal.pone.0192614 29438417 PMC5811218

[27] Vlachogiannis DM Xu Y Jin L González MC. Correlation networks of air particulate matter (PM_2.5_): a comparative study. Appl Netw Sci. (2021) 6:32. 10.1007/s41109-021-00373-8 33907706 PMC8062950

[28] Jia Y Rahn KA He K Wen T Wang Y. A novel technique for quantifying the regional component of urban aerosol solely from its sawtooth cycles. J Geophys Res. (2008) 113:D21309. 10.1029/2008JD010389

[29] Fisher RA. On the probable error of a coefficient of correlation deduced from a small sample. Metron. (1921) 1:3–32.

[30] Kenny DA. Statistics for the Social and Behavioral Sciences. Canada: Little, Brown and Company (1987). p. 407

[31] Granger CWJ. Investigating causal relations by econometric models and cross-spectral methods. Econometrica. (1969) 37:424–38. 10.2307/1912791

[32] Sfetsos A Vlachogiannis D. An analysis of ozone variation in the greater athens area using granger causality. Atmos Pollut Res. (2013) 4:290–7. 10.5094/APR.2013.032

[33] Global Modeling and Assimilation Office. MERRA-2 tavgM_2d_slv_Nx: 2d, Monthly mean, Time-Averaged, Single-Level, Assimilation, Single-Level Diagnostics, version 5.12.4. Greenbelt, MD: Goddard Space Flight Center Distributed Active Archive Center (GSFC DAAC) (2015).

[34] Stein A Draxler R Rolph G Stunder B Cohen M Ngan F. NOAA's HYSPLIT atmospheric transport and dispersion modeling system. Bull Am Meteorol Soc. (2015) 96:2059–77. 10.1175/BAMS-D-14-00110.1

[35] Carslaw DC Ropkins K. openair—an R package for air quality data analysis. Environ Model Softw. (2012) 27–28:52–61. 10.1016/j.envsoft.2011.09.008

[36] Shi C Nduka IC Yang Y Huang Y Yao R Zhang H . Characteristics and meteorological mechanisms of transboundary air pollution in a persistent heavy PM_2.5_ pollution episode in Central-East China. Atmos Environ. (2020) 223:117239. 10.1016/j.atmosenv.2019.117239

[37] LTP. Summary Report of the 4th stage (2013-2017) LTP Project. Incheon: National Institute of Environmental Research of South Korea (2019). Available online at: https://nier.go.kr/NIER/cmm/fms/NoLoginFileDown.do;jsessionid=C1A37B9309AC438907222958C0680EF9?atchFileId=FILE_000000000029154&fileSn=0

[38] Li J Yang W Wang Z Chen H Hu B Li J . A modeling study of source-receptor relationships in atmospheric particulate matter over Northeast Asia. Atmos Environ. (2014) 91:40–51. 10.1016/j.atmosenv.2014.03.027

[39] Kajino M Sato K Inomata Y Ueda H. Source-receptor relationships of nitrate in Northeast Asia and influence of sea salt on the long-range transport of nitrate. Atmos Environ. (2013) 79:67–78. 10.1016/j.atmosenv.2013.06.024

[40] Kajino M Ueda H Sato K Sakurai T. Spatial distribution of the source-receptor relationship of sulfur in Northeast Asia. Atmos Chem Phys. (2011) 11:6475–91. 10.5194/acp-11-6475-2011

[41] Lin M Oki T Bengtsson M Kanae S Holloway T Streets DG. Long-range transport of acidifying substances in East Asia-Part II: source-receptor relationships. Atmos Environ. (2008) 42:5956–67. 10.1016/j.atmosenv.2008.03.039

